# Prevention Research Centers: Contributions to Updating the Public Health Workforce Through Training

**Published:** 2005-03-15

**Authors:** Adele L Franks, Ross C Brownson, Elizabeth A Baker, Terry L Leet, Margret A O'Neall, Carol Bryant, Kelli McCormack Brown, Steven P Hooker, Dennis M Shepard, R. Pate Russell, Kathleen N Gillespie, Eduardo J Simoes

**Affiliations:** Michigan Center for Genomics & Public Health, University of Michigan, School of Public Health, Department of Epidemiology; Prevention Research Center, Saint Louis University School of Public Health, St Louis, Mo; Prevention Research Center, Saint Louis University School of Public Health, St Louis, Mo; Prevention Research Center, Saint Louis University School of Public Health, St Louis, Mo; Prevention Research Center, Saint Louis University School of Public Health, St Louis, Mo; Prevention Research Center, University of South Florida, Tampa, Fla; Prevention Research Center, University of South Florida, Tampa, Fla; Prevention Research Center, University of South Carolina, Columbia, SC; Prevention Research Center, University of South Carolina, Leesburg, SC; Department of Exercise Science, University of South Carolina, Columbia, SC; Department of Health Management and Policy, Saint Louis University School of Public Health, St Louis, Mo; Prevention Research Centers Program, Centers for Disease Control and Prevention, Atlanta, Ga

## Abstract

Because public health is a continually evolving field, it is essential to provide ample training opportunities for public health professionals. As a natural outgrowth of the Centers for Disease Control and Prevention's Prevention Research Centers Program, training courses of many types have been developed for public health practitioners working in the field. This article describes three of the Prevention Research Center training program offerings: Evidence-Based Public Health, Physical Activity and Public Health for Practitioners, and Social Marketing. These courses illustrate the commitment of the Prevention Research Centers Program to helping create a better trained public health workforce, thereby enhancing the likelihood of improving public health.

## Introduction

As exemplified by the genomics focus of this issue of *Preventing Chronic Disease*, public health is a continually evolving field. With an estimated 450,000 people working in salaried public health positions (1), many of whom received their training long ago, ensuring that members of this workforce receive the continuing education necessary to keep their competencies current is a daunting task. As public health incorporates new knowledge, changing methods, and technologic advances, regular training opportunities for public health professionals will become an ever greater necessity (2). While conferences such as the 19^th^ National Conference on Chronic Disease Prevention and Control, the featured abstracts of which are included in this issue, serve to acquaint participants with current matters, skill development usually requires more intensive training that includes active engagement. Therefore, making professional development opportunities and training courses more available to public health practitioners is essential (2).

In the 20 years since its inception, the Prevention Research Centers (PRC) program of the Centers for Disease Control and Prevention (CDC) has been offering training opportunities to public health practitioners in the United States and abroad. The PRCs are based on collaborations among schools of public health or medicine, communities, and public health agencies. The purpose of the collaborations is to design, test, and disseminate health promotion strategies that are effective in real-world settings. The PRCs' training programs have been a natural outgrowth of these collaborations, helping the PRCs carry out their mission to disseminate effective interventions. By training professionals and laypeople who work in the field, the PRCs help ensure that effective new strategies can be put in action for the benefit of communities. Because the prevention researchers themselves focus on applied community research, the training programs reflect their understanding of community needs, further increasing the value of the courses to public health professionals.

In keeping with the broad scope of the PRC program and the wide range of interests among the 28 centers, the PRCs provide many types of training (available from www.cdc.gov/prc/training/index.htm). For this issue of *Preventing Chronic Disease*, we highlight three training programs to illustrate their relevance to public health practitioners. The first, Evidence-based Public Health, provides a set of concepts and tools to help public health practitioners focus their scarce resources on efforts that stand the best chance of success, regardless of the health problem being addressed. The second, Physical Activity and Public Health for Practitioners, addresses the crucial public health problem of insufficient physical activity and teaches skills that public health practitioners can use to effectively mount physical activity promotion programs. The third, Social Marketing, provides training in a methodology that is insufficiently used in public health, despite the fact that it can increase the chance of success for nearly any health promotion effort.

## Evidence-Based Public Health

Public health practice is too often governed by short-term demands, management of crises, or long established habits. Practitioners' lack of sufficient training in more systematic approaches to priority setting and program selection serves to perpetuate a reactive style of public health practice. Recognizing the importance of cultivating a more systematic approach to the practice of public health, the Saint Louis University (SLU) PRC has developed a training course to increase the capacity of public health practitioners to find and use existing information and assessment tools in their daily work and to practice evidence-based public health. Evidence-based public health is a process that engages key stakeholders in "the development, implementation, and evaluation of effective programs and policies in public health through application of principles of scientific reasoning, including systematic uses of data and information systems and appropriate use of behavior science theory and program planning models" (3).

The Evidence-Based Public Health (EBPH) course was first developed in 1997 by the SLU School of Public Health and the Missouri Department of Health and Senior Services. It was later expanded in collaboration with the CDC, the Chronic Disease Directors, as well as the World Health Organization (WHO). In its current configuration, the EBPH course focuses on developing specific skills to improve public health practice. It introduces the basic concepts of evidence-based decision making and addresses how to use strategic planning processes and develop a concise statement of the issue under consideration, how to quantify the issue in accordance with basic principles of epidemiology and apply these principles to the available data, how to search the scientific literature and available databases to systematically review the evidence, how to assess the evidence and prioritize among options, how to develop a program action plan, and how to evaluate programs and policies after they are implemented.

The course is structured to encourage active engagement of students in practice exercises and case studies as well as in examination of ways the curriculum can be applied to the jobs they will be resuming after the training. The course is taught by multidisciplinary faculty that includes experts in epidemiology, behavioral science/health education, and economic evaluation. The course makes extensive use of online databases such as the Missouri Information for Community Assessment Web site (available from www.health.state.mo.us/MICA/nojava.html*), an interactive system that allows users to create a table of data from various data files including births, deaths, and hospital discharges.

Since 1997, this 2.5- to 4.5-day course has been offered 20 times and has reached more than 250 employees of local and state health departments in Missouri, approximately 145 public health practitioners from 36 other states, 80 practitioners in the Russian Federation, and 60 practitioners from Europe. Most of the participants have had no graduate training in public health. The course has been further adapted to meet the local needs and priorities of four states and three countries, and it has also been translated into Russian and Spanish. (For more information, including plans for future courses, contact Ross Brownson at brownson@slu.edu.)

The course was recorded in 2002, and a set of 16 CDs has been produced, with exercises and case studies for self-study. These materials are available  to people who cannot travel to a course location or prefer the self-study format (available from http://prc.slu.edu). In addition, *Evidence-Based Public Health*, a book published in 2003, now serves to augment the course for both students and teachers (4).

Course evaluations completed by course participants from 2001 to 2004 have shown very high levels of satisfaction with the course (8.5–10 on a 10-point scale) and with the instructors (8–10 on a 10-point scale). Nearly all participants (94%) have said that they expect to use their new skills in their daily work. Plans for the future include expanded formal follow-up of students to evaluate the impact of the course. Several universities and state health departments have initiated similar training courses for their personnel, indicating a recognized need for such courses.

## Physical Activity and Public Health for Practitioners

To halt the joint epidemics of obesity and diabetes in the United States, it is imperative to increase physical activity levels among people of all ages (5). Unfortunately, too few public health practitioners have the skills necessary to identify, implement, and evaluate evidence-based physical activity programs (6). To address these gaps in public health expertise, the University of South Carolina's Prevention Research Center developed an intensive six-day training course that has been offered annually since 1996.

The objectives of the course (the Physical Activity and Public Health [PAPH] Practitioner's Course on Community Interventions) are to give course participants the ability to 1) use public health and scientific information to identify and prioritize community-based physical activity interventions; 2) develop and implement community-based partnerships; 3) develop and implement both evidence-based individual behavioral interventions and policy/environmental interventions to promote physical activity; and 4) evaluate interventions to increase physical activity at the local level.

Each year, 25 participants are selected to take the course on the basis of their professional credentials, experience, and potential to enhance public health practice. Since the course began, 228 professionals representing 40 states and seven foreign countries have completed the training. These participants were affiliated with 21 colleges/universities; 31 state departments of health; and numerous other organizations such as hospitals, local health departments, nonprofit organizations, private foundations, research institutes, and federal government agencies. Faculty for the course consists of experts from the University of South Carolina's Arnold School of Public Health, the CDC, and other universities and nongovernmental organizations around the world.

During the 2001 Physical Activity and Public Health (PAPH) course, the rural estuarial town of Bluffton, SC, hosted a team project. The town was preparing to undergo rapid development, and the team saw an opportunity to expand the park system in a manner that could produce a continuous greenway from the historic downtown to the newly developing areas, thus encouraging foot and bicycle traffic as a means of transportation. The team's recommendations to the town included purchasing a privately held parcel of land that was home to an historic oyster-shucking factory. The team presented its recommendations to the community at a well-attended meeting in the local town hall. At that meeting, community groups interested in bicycle and walking trails interacted for the first time with the mayor and other town officials who made a public commitment to hold subsequent joint meetings. These meetings resulted in the formation of the Greater Bluffton Pathways Coalition, which soon became an active group with 150 members.

Primed to purchase the land, the town diligently pursued public funds available from the Beaufort County Open Land Trust. When the land became available for sale, the municipal leaders were ready to secure its purchase. Furthermore, the town was able to arrange for the historic oyster factory to remain in operation on the public land as an historical institution. Bluffton has come to trust the PAPH students as a valuable resource, providing knowledge and an objective perspective, and it continues to serve as an eager host community for the team exercises. The effort of Bluffton residents to increase green space and safe venues for physical activity and to maintain the historic character of their town continues along with the development process.

The program is designed to teach participants how to develop a logic model for evidence-based efforts to improve health through physical activity; how to select, plan and implement physical activity promotion programs in communities; how to measure outcomes; the importance of carefully planning and conducting a program evaluation; and the resources they will need to stay current with the latest developments in the field. The course uses multiple formats including didactic lectures with ample question and answer time, consultation sessions with experts, and a team field project. Course participants also have numerous opportunities for informal networking and individual tutorial sessions.

Local and state health departments and community organizations regularly enter into partnerships with the course organizers to provide opportunities for students to practice what they have learned in the classroom. These partners provide students with important information and insights into the features of the communities that serve as the settings for field projects. Results and recommendations from the field projects are in turn provided to community partners for potential implementation. Community members report having taken several actions on the basis of these recommendations, leading to environmental policy changes in the community (Sidebar).

To date, the course has been evaluated through participants' anonymous responses to a written questionnaire at the close of the course, as well as through exit interviews. Feedback from these efforts has led to a continuous process of course improvement.

The most recent postcourse evaluation results available (2003) showed a very high level of satisfaction among course participants and unanimous agreement that the course will influence their future professional activities. In open-ended questioning, respondents mentioned the opportunities for interaction with faculty and for networking with other participants as especially useful.

Longer-term evaluation based on how participants have subsequently used the training is currently under way. Meanwhile, follow-up surveys conducted by telephone and by mail to elicit feedback from participants in the first four years of the course (1996–1999) have yielded important information. Of the 63 respondents to the mailed survey, 65% reported an increase in their involvement in physical activity interventions after the course. Nearly all respondents (97%) said that they had applied information from the course in their daily work, 74% said that they had changed the way they plan and implement physical activity programs, and 75% said that they had increased their leadership role in physical activity promotion.

The success of this training program is perhaps best exemplified by its use as a model for developing other training programs such as the Nutrition and Public Health course sponsored by the CDC's Division of Nutrition and Physical Activity. The WHO Collaborating Center sponsored a similar course in Brazil last year in which 57 professionals were trained by an expert faculty from Brazil and around the world. Another course is planned for Colombia in 2005. In addition, WHO regularly sends participants to the United States course in hopes of improving their competencies and spawning interest around the world for physical activity promotion. Information about the course and application materials are available from www.prevention.sph.sc.edu/seapines/index.htm.

## Social Marketing

Social marketing involves the use of marketing principles in designing and implementing programs to promote socially beneficial behavior change (7-9). At its most basic level, it is a set of methods to increase understanding of a target audience so that practitioners can develop interventions most likely to resonate well with, and elicit the desired behavior change in, the intended audience. Its use as a tool in public health has been growing, although many in the field have incomplete understanding of its techniques, its proper use, or its potential benefits. To help public health professionals to acquire the knowledge, skill, motivation, and experience necessary to use social marketing effectively, the Florida Prevention Research Center (FPRC) at the University of South Florida offers several types of training courses to public health professionals working at the federal, state, and local levels.

To make both introductory training and continuing education in social marketing available, the FPRC offers the annual Social Marketing and Public Health Conference, Social Marketing in Public Health Field School courses, a Social Marketing in Public Health graduate certificate, and a variety of shorter training workshops tailored to meet the needs of a wide variety of public health practitioners.

The Social Marketing in Public Health Conference, which was initiated in 1991, has since attracted more than 3500 public health and social marketing practitioners from 50 states and 14 countries. The main conference is designed for professionals with some expertise in social marketing. To complement plenary sessions on current issues in the field, concurrent sessions feature case studies and analyses of emerging theoretical and methodological issues. The conference culminates in an array of half-day workshops on topics such as health message design principles and practice, formative research methods, low-cost materials development, pretesting techniques, and evaluation methods. The faculty consists of experts in social marketing from around the world (available from www.cme.hsc.usf.edu*.)

Just prior to the main conference, a two-day introductory training session is held to teach participants the basics of social marketing, including its conceptual framework, audience segmentation techniques, the use of formative research to develop a marketing plan, the development and pretesting of marketing materials and tactics, and the principles of program monitoring and evaluation. Participants work in teams, applying marketing concepts to specific public health problems. More than half of the participants in the introductory training session stay for the main conference as well.

The Social Marketing in Public Health Field Schools offer graduate-level courses taught by experts in social marketing, commercial marketing, and public health (available from www.cme.hsc.usf.edu). These courses are offered in an intensive five-day format. One course is offered just prior to, and one just after, the main conference in June to accommodate busy professionals who prefer a concentrated travel schedule. Another five-day course is offered in January. Two to four Field School courses have been offered each year, with enrollments varying from 28 to 110 (in 2004). Each of these five-day courses may be taken for three graduate credits by students enrolled in masters' degree programs or in the social marketing graduate certificate program (see below). Courses address strategic planning, formative research methods, media expertise, special communications skills (e.g., for low literacy and cross-cultural populations), and consumer behavior theory. Pretest–posttest evaluations of these training courses have consistently demonstrated a significant increase in participants' understanding of social marketing concepts and confidence in their ability to apply social marketing techniques.

To meet the growing need for advanced training and credentialing in social marketing and public health, the FPRC faculty has developed an 18-credit–hour graduate certificate program (available from www.outreach.usf.edu/gradcerts/certificates.asp) designed for experienced masters-prepared public health professionals. (Three have graduated to date.) The only such program for public health practitioners in the United States, the graduate certificate program requires students to complete six courses.

Each year, FPRC faculty members also provide intensive, multiday social marketing training workshops tailored to meet the needs of practitioners working in state and local public health departments. Since 2001, the FPRC has offered training in social marketing and community-based prevention marketing to state health departments in Alaska, Arkansas, California, Florida, Colorado, Idaho, Kentucky, North Carolina, Texas, and Missouri, as well as to numerous local health departments around the country. (Recent training workshops are available from http://publichealth.usf.edu/prc/training_matrix.pdf.)

Other social marketing resources relevant to public health practitioners are available from http://publichealth.usf.edu/prc/training.html and from http://turningpointprogram.org?Pages/socialmakt.html.

## Summary

A well-trained public health workforce is essential if public health research is to have a tangible impact on populations. The three PRC-generated training courses highlighted here illustrate the commitment of the PRC program to improving public health, not only through innovative research with communities, but through direct training of public health practitioners (more offerings are available from www.cdc.gov/prc/training/index.htm).

The EBPH and PAPH courses build on the successful application of evidence-based approaches in clinical disciplines (10,11), the increased availability of online data, and outstanding new systematic reviews such as the *Guide to Community Preventive Services* (12,13) (available from www.thecommunityguide.org) that seek to improve the use of scientific information in public health practice settings. Social marketing training offers techniques to help public health practitioners to better understand their target audiences so that public health efforts to change behavior have the best chance of success. These three training courses developed and offered by PRCs constitute a meaningful contribution to the ongoing training of public health practitioners and, by creating a better trained workforce, enhance the likelihood that public health interventions are selected for implementation on the basis of scientific evidence of effectiveness and that the interventions selected are ultimately successful.
